# Presence of anti-nuclear antibodies is a risk factor for the appearance of anti-drug antibodies during infliximab or adalimumab therapy in patients with rheumatoid arthritis

**DOI:** 10.1371/journal.pone.0243729

**Published:** 2020-12-14

**Authors:** Ayano Mori, Toshiharu Saito, Miho Takahashi, Miho Shibata, Goh Tsuji, Saori Hatachi, Soshi Takahashi, Shunichi Kumagai

**Affiliations:** 1 The Shinko Institute for Medical Research, Shinko Hospital, Kobe, Japan; 2 The Center for Rheumatic Disease, Shinko Hospital, Kobe, Japan; Nippon Medical School, JAPAN

## Abstract

This study aimed to directly analyze the potential relationship of anti-nuclear antibodies (ANA) before and after the administration of TNF-α inhibitors (TNFi) with the appearance of anti-drug antibodies (ADrA) in patients with rheumatoid arthritis (RA). A total of 121 cases, viz., 38, 53, and 30 cases treated with infliximab (IFX), adalimumab (ADA), and etanercept (ETN), respectively, were enrolled. The ANA titers were measured using indirect immunefluorescence assay (IF-ANA) and multiplex flow immunoassay (ANA Screen) before and serially during the therapy. The anti-IFX antibodies (HACA) and anti-ADA antibodies (AAA) were measured with a radioimmunoassay. ADrA turned positive in 14 (36.8%) among 38 patients treated with IFX, and 16 (30.2%) among 53 treated with ADA. All of them were positive for IF-ANA before TNFi administration, while ADrA never appeared in any of the 15 patients negative for IF-ANA (< 40). IF-ANA of high titers (≥ 320 and ≥ 640) before IFX treatment showed a significant association with the appearance of HACA 52 weeks after IFX (*P* = 0.040 and 0.017, respectively), whereas AAA appearance was not related to IF-ANA titers before treatment. Moreover, IF-ANA of high titers before IFX treatment was significantly associated with inefficacy and discontinuation of the treatment. The positivity of anti-SS-A antibodies before therapy might be a risk factor for ADrA appearance in patients treated with IFX or ADA. The percentage of patients whose IF-ANA titers increased was significantly higher with IFX than with ADA or ETN treatments (*P* = 0.026 and 0.022, respectively). High ANA titers and positive ANA Screen after IFX therapy showed a significant association with HACA appearance and possibly led to treatment failure. Among the three TNFi, only IFX showed a close relationship with IF-ANA and ADrA appearance, suggesting the interaction of immunogenicity with autoimmunity as well as the advantage of ANA measurement before TNFi therapy.

## Introduction

TNF-α inhibitors (TNFi) such as infliximab (IFX), adalimumab (ADA), etanercept (ETN), golimumab, and certolizumab pegol are dramatically effective for the treatment of rheumatoid arthritis (RA). However, a certain proportion of RA patients do not respond well to TNFi from the beginning (primary failure) or lose treatment efficacy following an initially good response (secondary failure) [1]. These biologics have been shown to have immunogenicity and often generate anti-drug antibodies (ADrA) against TNFi such as anti-IFX antibody (HACA: human anti-chimeric antibodies) and anti-ADA antibodies (AAA) [2]. The incidence of ADrA varies considerably with the measurement method, observation period, dosage, and age; 7% to 53% has been observed in IFX, 1% to 31% in ADA, and few in ETN [1–3]. Appearance of ADrA not only leads to drug-induced allergic reactions in the patients but also to secondary failure associated with undetectable serum concentrations of the biologics [1,3–5]. To date, there is no way to distinguish a non-responder from a responder before commencing treatment with a biologic [1]. Most TNFi have an additional adverse effect, which is the induction of systemic autoimmune diseases such as systemic lupus erythematosus (SLE) and systemic vasculitis during the TNFi therapy [6–8]. Autoantibodies such as anti-nuclear antibodies (ANA) and anti-double-stranded (ds) DNA antibodies (dsDNA Ab) can be newly induced (seroconversion) or increase their titers during anti-TNF therapy [9,10].

Regarding the relationship of autoimmune disease induction and ANA to clinical response of TNFi therapy, Yukawa et al. showed that increases in the titers of immunofluorescent ANA (IF-ANA) and dsDNA Ab after IFX therapy were related with poor clinical response and that high titers of ANA (≥ 160) before IFX therapy were also related with poor clinical response [11]. Takase et al. also described that 4 cases (5.4%) out of 74 primary failures and 24 cases (21.6%) out of 111 secondary failures among the 454 patients became IF-ANA positive after TNFi treatment [9]. Ishikawa et al. reported that IF-ANA development (≥2 times the baseline levels) during IFX as well as other TNFi could be a marker of poor clinical response to the therapy in RA patients [12]. In contrast, Hoxha et al. showed that AAA could be an early marker associated with poor clinical response in 58 rheumatic patients treated with ADA; however, ANA positivity was not significantly different between AAA-positive and negative groups [13]. Jani et al. reviewed drug safety and immunogenicity of TNFi and mentioned that a proportion of patients on TNFi may develop ANA and dsDNA Ab due to immunogenicity, indicating a linkage of autoantibody production (autoimmunity) with development of ADrA (immunogenicity) and probably with treatment failure [14]. They also described that patients predisposed to developing immunogenicity may also be prone to seroconversion of other autoantibodies, yet few studies have been performed to clarify the direct relationship of ANA with ADrA appearance and clinical response in RA patients with long-term TNFi treatment.

Therefore, the aim of this study was to analyze the direct relationship of ANA presence before and after the administration of IFX or ADA with the appearance of ADrA in order to evaluate the usefulness of ANA measurement for predicting treatment inefficacy and secondary failure in RA patients.

## Materials and methods

### Patients and the treatment

A total of 163 adult RA patients were newly introduced to one of the three kinds of TNFi (46, 67, and 50 cases for IFX, ADA, and ETN, respectively) at the Shinko Hospital, Kobe, Japan, from 2011 to 2018. All patients met the 2010 ACR/EULAR rheumatoid arthritis classification criteria. Patients were treated with IFX at 3 mg/kg intravenously at week 0, 2, and 6, and 3–10 mg/kg every 6–10 weeks thereafter, with ADA at 40–80 mg every 2 weeks subcutaneously, or with ETN at 25–50mg per week subcutaneously; the dose and interval were changed at the discretion of the physician. Fifteen cases were excluded due to non–agreement with the study, and thirteen cases were excluded due to discontinuation of the treatment within 1 year because of death, financial reasons, operation, hospital transfer, or pregnancy. In addition, fourteen cases were due to discontinuation of IFX within 14 weeks, ADA within 8 weeks, and ETN within 8 weeks. As a result, a total of 121 patients treated with TNFi (38, 53, and 30 cases for IFX, ADA, and ETN, respectively) were prospectively enrolled in this study. As shown in Table 1, baseline characteristics of the patients enrolled were similar among the three treatment groups except for the frequency of concomitant use of methotrexate (MTX). Mean age (SD) of the patients excluded from our study (IFX, 8; ADA, 14; ETN, 20) was 47.4 (19.4), 62.4 (12.6), 53.1 (18.0), respectively, and the percentage of female was 87.5%, 85.7%, 95.0%, respectively. There were no demographic differences between our participants and those excluded, suggesting no selection bias in our participants. However, our sample was not representative of a larger population because of its small size.

**Table 1:** Characteristics of 121 RA patients.

	All patients	IFX	ADA	ETN	*P*-value^a^
	(n = 121)	(n = 38)	(n = 53)	(n = 30)	
Age, mean (SD), years old	52.4 (16.4)	48.3 (16.3)	54.5 (13.7)	53.9 (20.3)	0.230
Females, n (%)	103 (85.1)	36 (94.7)	41 (77.4)	26 (86.7)	0.065
Symptom duration, mean (SD), years	7.4 (8.4)	9.3 (9.2)	5.7 (7.9)	7.4 (7.8)	0.072
DAS28-CRP, mean (SD)	3.8 (1.1)	3.9 (1.0)	3.7 (1.1)	4.0 (1.0)	0.302
RF positive, n (%)	93 (76.9)	28 (73.7)	39 (73.6)	26 (86.7)	0.413
Anti-CCP antibody positive, n (%)	99 (81.8)	32 (84.2)	41 (77.4)	26 (86.7)	0.572
IF-ANA positive, n (%)	101 (83.5)	32 (84.2)	44 (83.0)	25 (83.3)	1.0
Concomitant sDMARDs	110 (90.9)	38 (100)	48 (90.6)	24 (80.0)	0.006
MTX, n (%)	103 (85.1)	38 (100)	47 (88.7)	18 (60.0)	< 0.001
MTX dosage, mean (SD), mg/week	8.7 (3.8)	9.4 (4.3)	8.5 (3.7)	7.9 (2.5)	0.724
As a 1st biologic, n (%)	103 (85.1)	32 (84.2)	42 (79.2)	29 (96.7)	0.074
PSL users, n (%)	63 (52.1)	17 (44.7)	25 (47.2)	21 (70.0)	0.077
PSL dosage, mean (SD), mg/day	4.8 (2.9)	4.8 (2.4)	5.4 (2.5)	5.1 (3.3)	0.196
Other autoimmune diseases, n (%)	5 (4.1)	0 (0)	4 (7.5)	1 (3.3)	0.207

^a^For differences among three groups (IFX vs. ADA vs. ETN), Kruskal-Wallis tests were used for non-normally distributed continuous variables and Fisher's exact tests were used for dichotomous variables.

IFX, infliximab; ADA, adalimumab; ETN, etanercept; DAS28-CRP, Disease Activity Score in 28 joints using C-reactive protein; RF, rheumatoid factor; anti-CCP, anti-cyclic citrullinated peptide; ANA, anti-nuclear antibodies; IF-ANA, immunofluorescent-ANA, sDMARD, synthetic disease modifying anti-rheumatic drugs; MTX, methotrexate; PSL, prednisolone.

There were no patients with other autoimmune diseases in the IFX group, although the ADA group contained two patients with Sjogren's syndrome, one patient with mixed connective tissue disease (MCTD), and one patient with SLE. One patient in the ETN group had Sjogren's syndrome. There were no new cases of SLE onset during anti-TNF therapy, however, one patient with SLE in the ADA group had been in remission during the ADA administration.

### Observation period and the details of TNFi treatment

The observation period for our patients was 52 weeks fundamentally; however, the period for patients treated with IFX was extended to 156 weeks because HACA was often detected after 52 weeks. We also measured AAA for ADA patients after 52 weeks, and added the data that showed change of IF-ANA titers and the existence of AAA until 156 weeks for only 12 patients treated with ADA. Therefore, analysis for ADA patients was confined to within 52 weeks because the data after 52 weeks potentially contained selection bias.

IFX, ADA, and ETN were usually started at 3mg/kg, 40 mg every 2 weeks, and 50 mg weekly, respectively. IFX dosage of the last observation at 156 weeks was 3mg/kg, 3.1–6 mg/kg, 6.1–9.9 mg/kg, and 10 mg/kg in 9, 13, 1, and 15 patients respectively; intervals of IFX administration were ≤7 weeks, 8 weeks, and ≥9 weeks in 6, 30, and 2 patients, respectively. The last dosage of ADA was 40 mg every 2 weeks, 40mg every 4 weeks, and 80 mg every 2 weeks in 45, 3, and 5 patients, respectively. The last dosage of ETN was 25 mg weekly in 4 patients and 50 mg weekly in 26 patients during the therapy.

### Evaluation of efficacy and treatment discontinuation

Disease activity score in 28 joints using the C-reactive protein (DAS28-CRP) was used to evaluate disease activity proposed by the European League against Rheumatism (EULAR) [15]. Poor response (inefficacy) to the therapy was judged at the physician's discretion by EULAR response criteria based on DAS28-CRP, and the treatment was discontinued by the physician when inefficacy or adverse effects were observed.

### Measurement of ANA

IF-ANA was measured serially over time with a computer-aided immunofluorescence microscopy system (EUROPattern Cosmic IFA analyzer from EUROIMMUN, Luebeck, Germany) using a specific reagent, Premmune HEp20-10 IIF-ANA "Cosmic" (EUROIMMUN) [16]. EUROPattern detects more than 100 kinds of ANA with high sensitivity but with low specificity for systemic rheumatic diseases. Sera were diluted in 2-fold serial dilutions and IF-ANA titers were measured semi-quantitatively from 40-fold to 5,120-fold. Positivity was determined if the titer was 40 or over. An increase or a decrease in IF-ANA titers was defined as more than 2-times change from baseline values or onset of seroconversion.

In addition, the ANA Screen, which detects 11 components of disease-specific ANA (dsDNA [IgG class], chromatin, Sm, RNP/Sm, RNP, SS-A, SS-B, Scl-70, centromere B, ribosomal protein, and Jo-1) with high specificity, was conducted with multiplex flow immunoassay, BioPlex ANA screen (Bio-Rad, Hercules, CA) using BioPlex2200 system from Bio-Rad [10]. The ANA Screen was determined to be positive if one or more of tests for the disease-specific ANA in the panel were positive.

### Measurement of ADrA to IFX and ADA, and the drug levels

The serum concentrations of HACA and AAA as well as their serum drug levels were measured over time with a radioimmunoassay (RIA) or an enzyme-linked immunosorbent assay (ELISA) at Sanquin (Amsterdam, Netherlands) in 38 and 53 patients treated with IFX and ADA, respectively [1,3]. We did not measure anti-ETN antibodies because of their previously reported rare appearance, which would have made the statistical analysis between ADrA-positive and negative groups challenging [1–3]. ADrA measurement was performed at least twice (22 and 46 weeks in principal) for the IFX-treated group, or at 4, 12, 24, and 48 weeks in principal for the ADA-treated group. The measurement was added when the efficacy of therapy was diminished or when the therapy was discontinued because of adverse events such as infusion reaction. The drug levels of IFX and ADA were also measured at the same time.

### Statistics and ethics

Data were compared among two- or three groups using Kruskal-Wallis tests, Mann-Whitney's U tests, Fisher's exact tests, and Wilcoxon signed rank tests as deemed appropriate. Simple logistic regression analysis was performed to examine the association between IF-ANA titers and ADrA appearance or treatment inefficacy or discontinuation. The tests were two-tailed and a value of *P <*0.05 was considered significant. All statistical analyses were performed using JMP®10 (SAS Institute Inc., Cary, NC). This study was carried out under approval of the ethics committee of Shinko Hospital and all patients agreed to participate in the study (approval numbers: 947, 1306, 1743).

## Results

### Appearance of ADrA and treatment discontinuation during anti-TNF therapy

**Fig 1:**
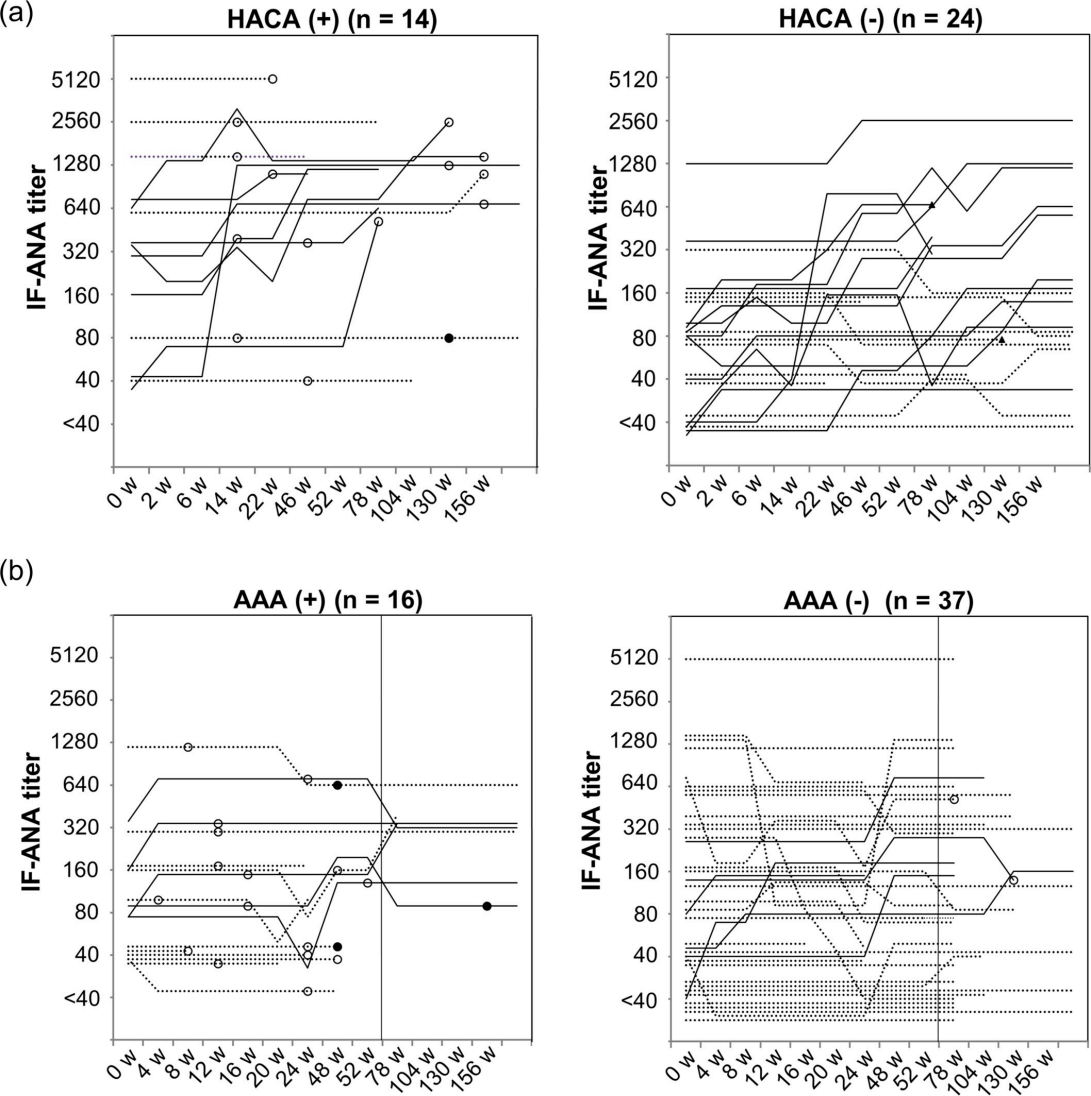
Change of IF-ANA titers and the presence of ADrA in patients treated with anti-TNF inhibitors. IF-ANA titers in the patients with (left) or without (right) ADrA against (a) IFX or (b) ADA are presented in the line graphs. Solid lines indicate the patients whose IF-ANA titers at the endpoint of the observation increased as compared with that of the starting point. Dashed lines indicate the patients whose titers were stable or decreased. Interruption of the graph indicates withdrawal of the anti-TNF therapy. The white circle (○) indicates the time when ADrA turned positive, and the black circle (●) indicates when ADrA turned negative again. The black triangles (▲) indicate the patients who discontinued the IFX therapy due to hospital transfer or financial reasons between 53–156 weeks (w). ADrA, anti-drug antibodies; IFX, infliximab; ADA, adalimumab; HACA, anti-IFX antibodies; AAA, anti-ADA antibodies.

Changes in IF-ANA titers of 38 patients treated with IFX and 53 patients treated with ADA are shown in Fig 1A and 1B, respectively. The figures for patients who became ADrA-positive are separately shown from ADrA negative patients; the timepoint where ADrA firstly appeared is marked with open circles (left sides of Fig 1A and 1B). HACA appeared 14 weeks after IFX therapy in the earliest case and became positive in 8 cases (21.1%) within 1 year (52 weeks), 11 cases (28.9%) within 2 years (104 weeks), and 14 cases (36.8%) within 3 years (156 weeks) (Fig 1A). On the other hand, AAA appeared in 16 of 53 cases (30.2%) within 52 weeks of ADA therapy (Fig 1B). AAA became positive as early as 4 weeks after ADA therapy, and the mean time for AAA appearance was 21.7 weeks which was significantly faster than 67.6 weeks for HACA appearance (*P* = 0.009).

The serum concentration of IFX at the point of HACA appearance in 14 patients was less than 0.1 μg/ml except for one patient with 0.3 μg/ml IFX. The levels were significantly low compared with the trough concentration of 5.9 ± 6.5 μg/ml (mean ± SD) in 24 patients negative for HACA (*P* < 0.0001). The serum concentration of ADA at the point of AAA appearance in 16 patients was less than 0.1 μg/ml in six patients and 0.6–14.1 μg/ml in other ten patients; the levels were significantly low as compared with the trough concentration of 13.2 ± 9.2 μg/ml (mean ± SD) in 37 patients negative for AAA (*P* < 0.0001).

As for treatment discontinuation, 13 (24.5%) among 53 treated with ADA discontinued the therapy within 52 weeks (Fig 1B), whereas only 5 cases (13.2%) discontinued IFX within 52 weeks and the number increasing to 18 cases (47.4%) within 156 weeks (Fig 1A). In both IFX and ADA treatment groups, the discontinuation was observed more frequently in the ADrA positive group. The rate of treatment inefficacy and discontinuation between 0–52 weeks was higher in the HACA-positive group than the negative group (5/8 [62.5%] vs. 3/30 [10.0%], *P* = 0.0049; and 3/8 [37.5%] vs. 2/30 [6.7%], *P* = 0.053, respectively), and the rate at 156 weeks was also significantly higher in HACA-positive group than in negative (8/14 [57.1%] vs. 4/22 [18.2%], *P* = 0.029; and 11/14 [78.6%] vs. 5/22 [22.7%], *P* = 0.002, respectively). The rate of inefficacy and discontinuation of ADA treatment within 52 weeks was also higher in the AAA-positive group than in the negative group (14/16 [87.5%] vs. 5/37 [13.5%], *P* < 0.0001; and 8/16 [50.0%] vs. 5/37 [13.5%], *P* = 0.012, respectively).

### Relation of ANA presence before anti-TNF therapy to ADrA appearance

**Fig 2:**
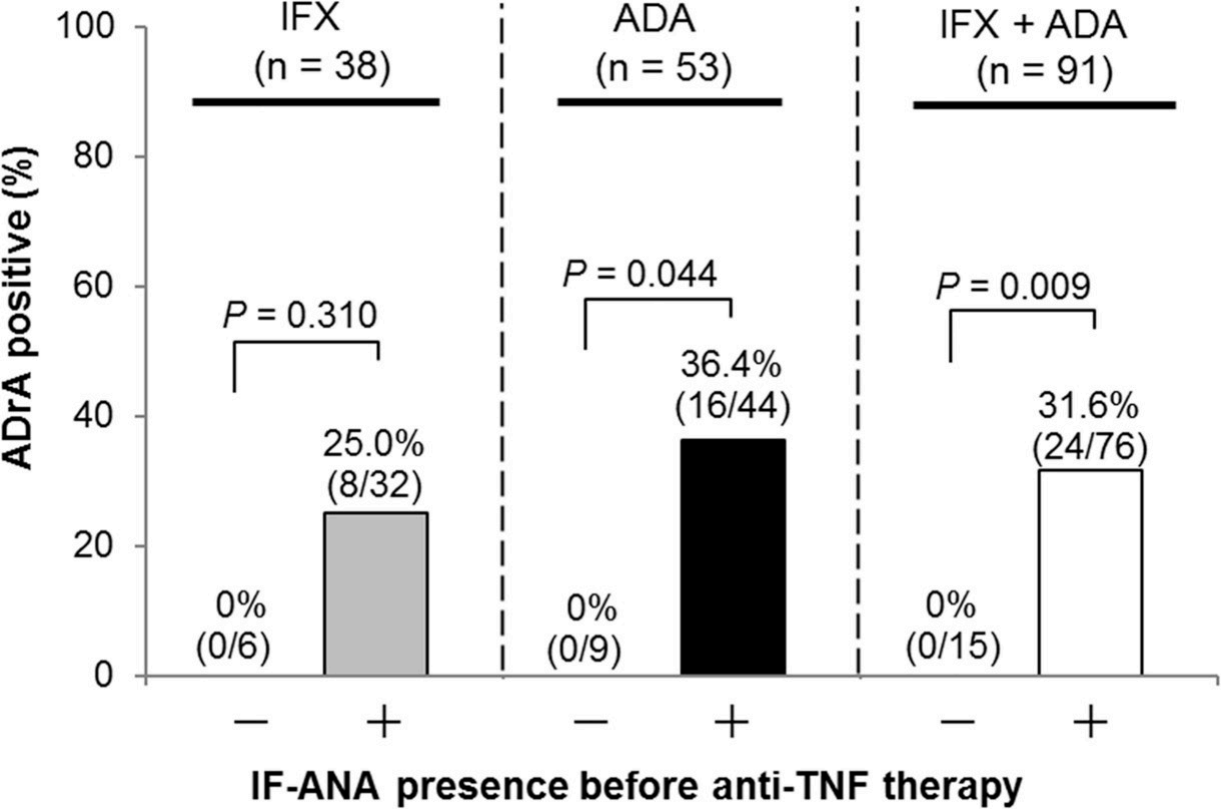
ADrA did not appear in the patients negative for IF-ANA before starting anti-TNF therapy. The rate of ADrA positive between 0–52 weeks was compared by IF-ANA presence or absence before anti-TNF therapy. The percentages and absolute numbers of each group of patients are indicated just above the bar graphs. Fisher’s exact test was used for comparison. ADrA, anti-drug antibodies; IFX, infliximab; ADA, adalimumab.

The positive rate for IF-ANA before therapy was 84.2% (32 of 38 patients), 83.0% (44 of 53 patients), and 83.3% (25 of 30 patients) in IFX, ADA, and ETN groups, respectively. The positive rate for ANA Screen before therapy was low (IFX, 23.7%; ADA, 34.0%; ETN, 13.3%); however, the positive rate in both IF-ANA and ANA Screen was not significantly different among the three groups. There were two cases positive for dsDNA Ab before therapy only in the ADA group. Following this, the relationship of ANA presence before TNFi therapy with the appearance of ADrA was investigated in 38 IFX-treated patients including 14 HACA-positive cases and in 53 ADA-treated patients including 16 AAA-positive cases. Notably, ADrA never appeared in 15 patients negative for IF-ANA before TNFi (6 cases with IFX and 9 cases with ADA), whereas IF-ANA was positive (≥ 40) before TNFi in all of the patients positive for ADrA (Fig 2). The positive rate for ADrA was significantly higher in the IF-ANA-positive group before TNFi than in the negative group (*P* = 0.009). The positive rate of AAA during the 52 weeks was significantly higher in patients positive for IF-ANA before ADA than in those negative (16/44 [36.4%] vs. 0/9 [0%], *P* = 0.044), whereas that of HACA during the 52 weeks was high but not significant in patients positive for IF-ANA. Notably, HACA never appeared until 156 weeks in 6 cases negative for IF-ANA before IFX. Positive likelihood ratio (LR+) of IF-ANA for appearance of ADrA was 1.29 (not significant) whereas the negative likelihood ratio (LR-) was zero, and the odds ratio (OR) was calculated as infinitely great (95% CI = 3.2– ∞, *P* = 0.0014), although the sample size was small. There was no relationship between the IF-ANA staining patterns (homogeneous, speckled, nucleolar, and cytoplasmic) before therapy and ADrA appearance.

Positivity for ANA Screen before the therapy was not significantly related to ADrA appearance, although the rate of the appearance had tended to increase in the patients positive for ANA Screen (40.7% vs 20.3%, *P* = 0.067) (S1 Fig). However, LR+ and LR- of the ANA Screen before the treatment were 1.91 and 0.71, respectively, and OR was calculated as 2.7 (95% CI = 1.01–7.26, *P* = 0.048). Relationship of ANA Screen and eleven components of disease-specific ANA before TNFi therapy to the appearance of ADrA during 0 to 52 weeks is shown in S2 Fig. LR+ and LR- of the SSA Ab before the treatment were 2.44 and 0.80, respectively, and OR was calculated as 3.04 (95% CI = 0.94–9.70, *P* = 0.062). In this study, SSA Ab was positive in 15 (55.6%) of 27 patients positive for ANA Screen before anti-TNF therapy.

In summary, the presence of IF-ANA and probably disease-specific ANA before anti-TNF therapy were closely related to ADrA appearance, suggesting an association of immunogenicity with autoimmunity.

### Changes in ANA positivity and their titers during TNFi treatment

**Fig 3:**
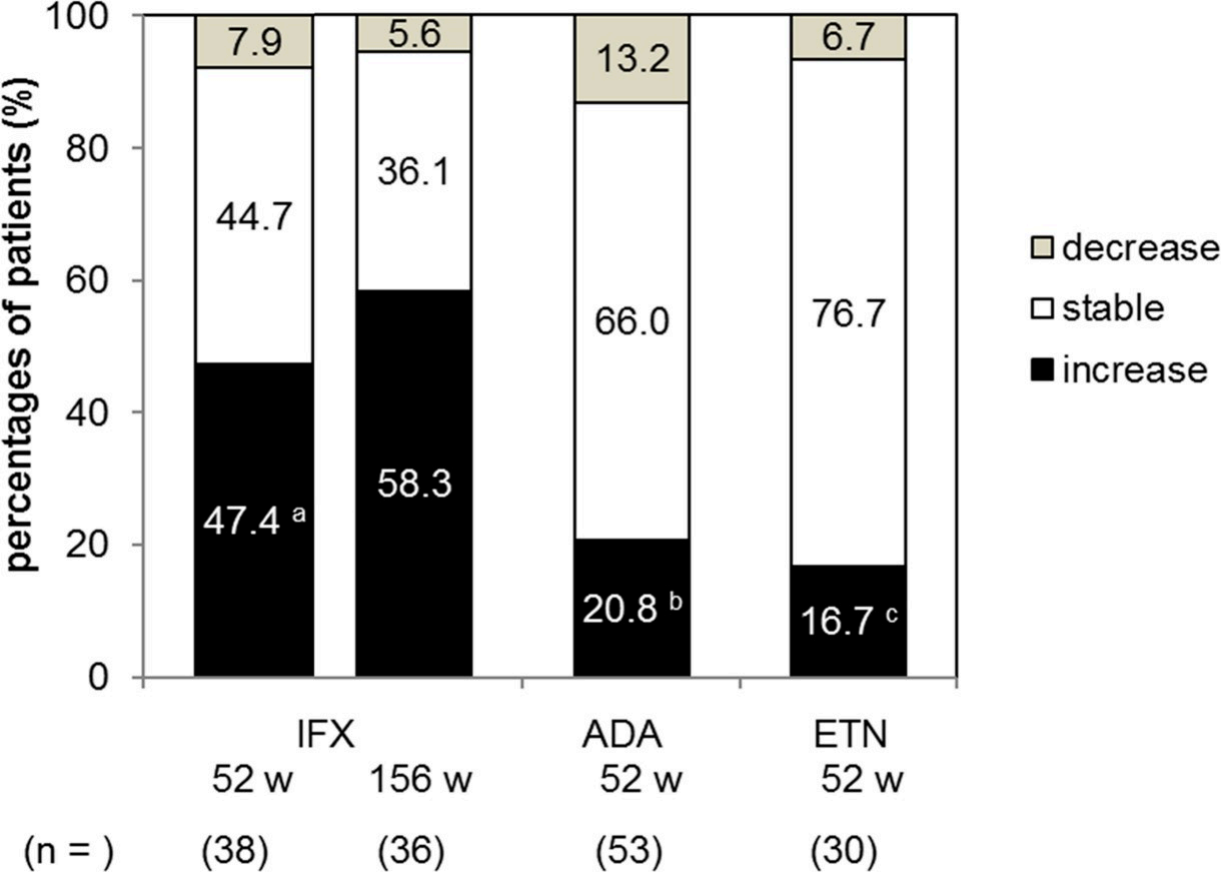
Changes in the titers of IF-ANA after each anti-TNF therapy. Percentages of patients whose IF-ANA titers increased, were stable, and decreased after each anti-TNF therapy (IFX, 0–52 weeks or 0–156 weeks; ADA, ETN, 0–52 weeks) are shown in the bar graph. The IF-ANA change was classified into 3 groups as increase, no change (stable), or decrease in IF-ANA value after the therapy compared with the value before the therapy. IF-ANA increase or decrease was defined as more than 2 times increase or decrease from baseline values, or onset of positive- or negative-seroconversion. IFX, infliximab; ADA, adalimumab; ETN, etanercept. w, weeks. *P* < 0.05; a vs. b, c.

Next, in order to compare the intensity of autoimmunity caused by the 3 kinds of TNFi, changes in IF-ANA titers and anti-dsDNA antibodies were compared from the start of TNFi until the end of the observation period. IF-ANA titers increased in 18 (47.4%) of 38 cases treated with IFX, and the percentage of patients whose IF-ANA titers increased was significantly higher in the IFX group than in the ADA- and in ETN groups (*P* = 0.026 and *P* = 0.022, respectively) (Fig 3). Four cases in the IFX group, 1 case in the ADA group, and 1 case in the ETN group turned positive for IF-ANA after therapy (seroconversion), whereas no cases in the IFX group, 2 cases in the ADA group, and 1 case in the ETN group turned negative for IF-ANA after therapy. There were no cases of lupus onset in all 121 cases during the observation time.

As for ANA Screen, only 5 cases (IFX, 2; ADA, 2; ETN, 1) turned positive from negative; however, the positive rate remained nearly unchanged after the therapy. As for dsDNA Ab, only 3 patients (IFX, 1; ADA, 2; ETN, 0) turned positive from negative after the therapy, although the mean titers slightly but significantly increased after treatment with IFX, ADA, and ETN (1.42 to 2.47 IU/ml, *P* = 0.0005; 2.43 to 3.92 IU/ml, *P* < 0.0001, and 1.40 to 1.93 IU/ml, *P* = 0.0023, respectively) (S3 Fig).

### Relationship of IF-ANA titers after IFX therapy with ADrA appearance

**Fig 4:**
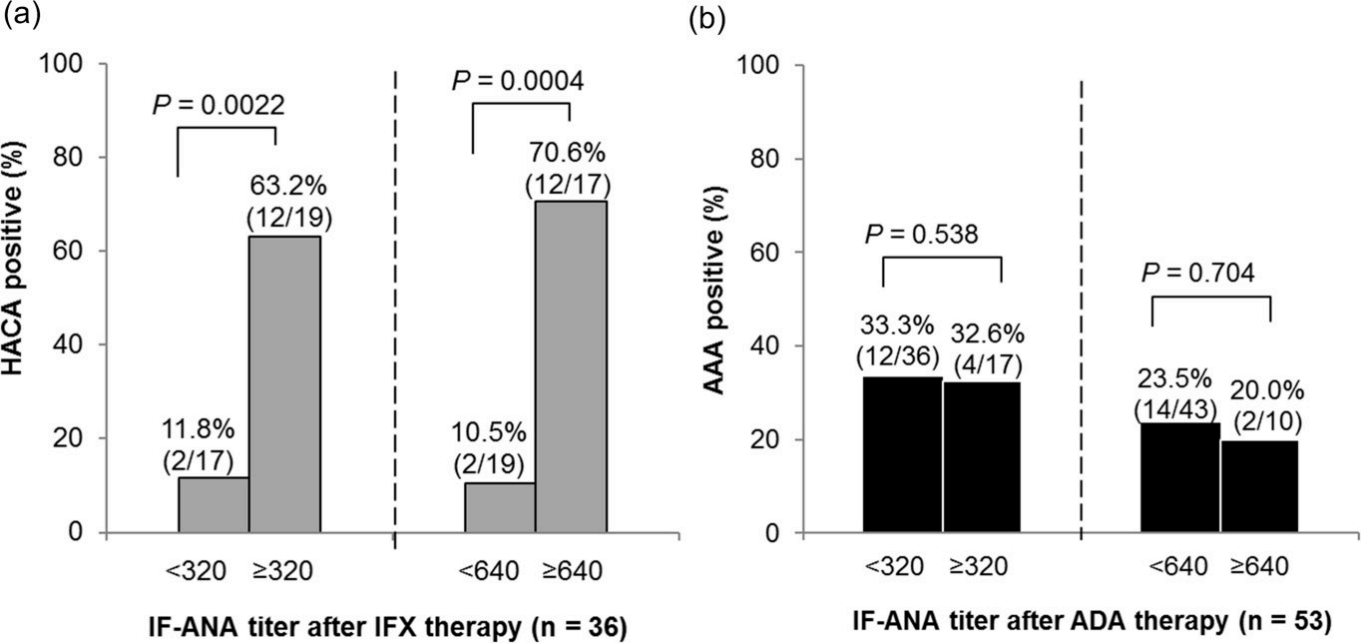
High titer of IF-ANA after IFX but not ADA therapy was related with ADrA appearance. (a) The rate of HACA positive between 0–156 weeks was compared between IF-ANA titer after IFX of <320 and ≥320/ <640 and ≥640. (b) The rate of AAA positive between 0–52 weeks was compared between IF-ANA titer after ADA of <320 and ≥320/ <640 and ≥640. The percentages and absolute numbers of each group of patients are indicated just above the bar graphs. Fisher’s exact tests were used for comparison. IFX, infliximab; ADA, adalimumab; ADrA, anti-drug antibodies; HACA, anti-IFX antibodies; AAA, anti-ADA antibodies.

As seen in Fig 1, IF-ANA titers seemed to increase after IFX therapy, especially in the HACA-positive group, which often resulted in treatment discontinuation. As to the relationship of IF-ANA titers to ADrA appearance after IFX and ADA, the positive rate for HACA was significantly higher in the patients positive for IF-ANA titers of ≥320 and ≥640 after the therapy (*P* = 0.0022 and *P* = 0.0004, respectively); however, IF-ANA titers after ADA therapy were not related to AAA appearance (Fig 4). The rate of ADrA appearance was higher in the patients whose IF-ANA had increased after therapy than in those not increased (43.8% vs. 28.1%) but the difference was not significant (S4 Fig).

As to ANA Screen, the positive rate of ADrA was significantly higher in the patients positive for ANA Screen than in the negative after both IFX for 0–156 weeks and ADA therapy for 0–52 weeks (8/11 [72.7%] vs. 6/25 [24.0%], *P* = 0.0097; and 9/17 [52.9%] vs. 7/36 [19.4%], *P* = 0.023, respectively). The mean titers of dsDNA Ab significantly increased more in the patients positive for HACA than in those negative (*P* = 0.022) (S5 Fig), but the increase of dsDNA Ab was not different between AAA presence or absence. These results suggested that the association between autoimmunity and immunogenicity was possibly stronger in patients treated with IFX than those with ADA.

### Prediction of ADrA appearance, treatment inefficacy, and discontinuation by IF-ANA titers

**Fig 5:**
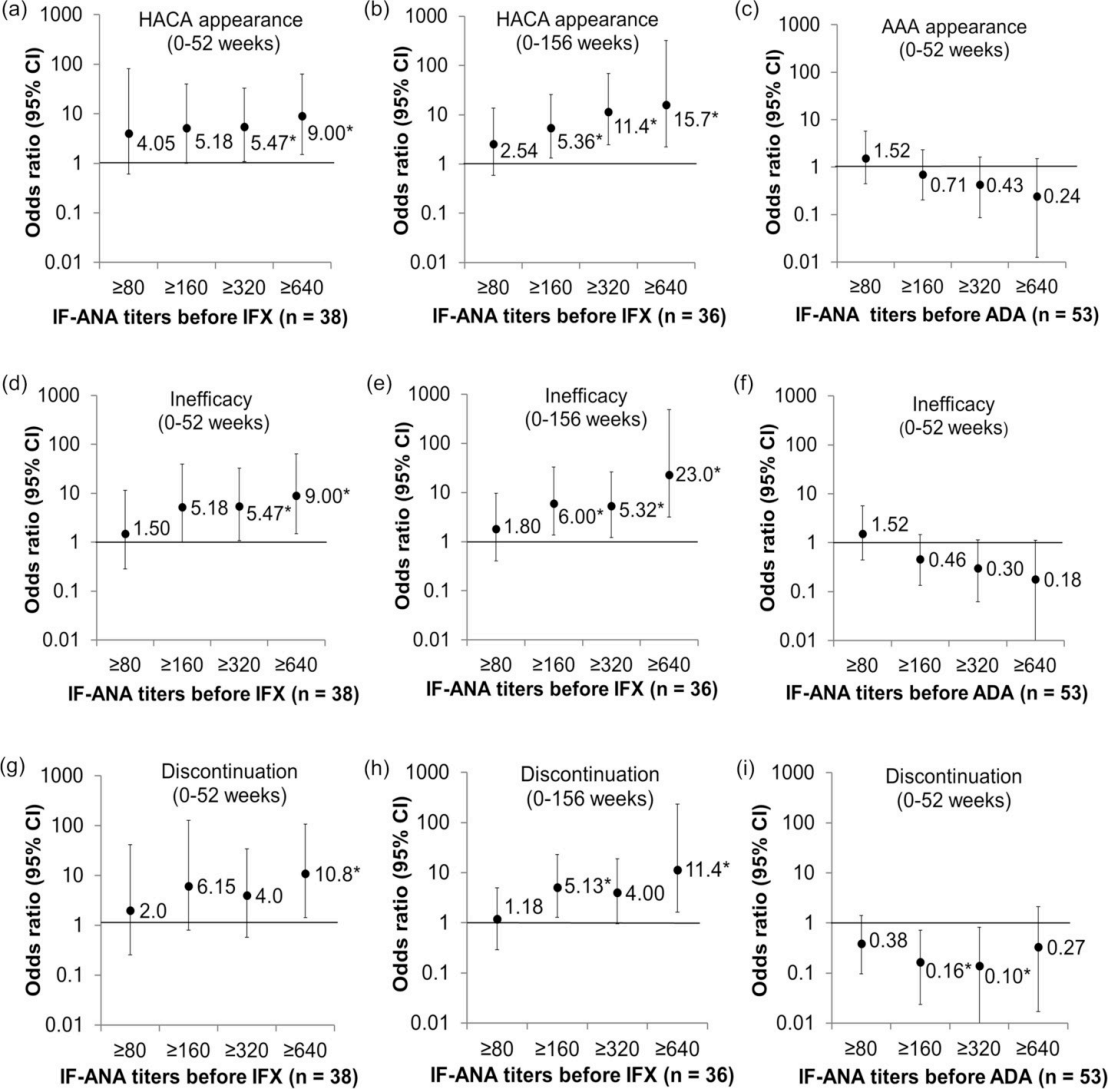
IF-ANA titers before IFX therapy were associated with HACA appearance and the treatment effect. Odds ratios for each outcome were calculated using simple logistic regression models. Simple model of HACA appearance between 0–52 weeks (a) or between 0–156 weeks (b) in the patients positive for IF-ANA titers of ≥80, ≥160, ≥320, ≥640 before starting IFX therapy. Simple model of AAA appearance between 0–52 weeks in the patients positive for IF-ANA titers of ≥80, ≥160, ≥320, ≥640 before starting ADA therapy (c). The same analyses as for (a)–(c) were performed for treatment inefficacy in (d)–(f), and for treatment discontinuation in (g)–(i). In (b), (e), and (h), two patients treated with IFX were excluded from the analysis due to discontinuation by hospital transfer or financial reasons. Mean values are indicated by dots with numbers, and 95% confidence intervals are indicated by two-sided lines. HACA, anti-IFX antibodies; AAA, anti-ADA antibodies. *, *P* < 0.05.

Finally, in order to reveal whether measuring IF-ANA enables us to predict treatment failure through estimation of ADrA appearance, we analyzed the relationship of IF-ANA before TNFi therapy to treatment inefficacy and discontinuation in addition to ADrA appearance. The rate of treatment inefficacy and discontinuation within 52 weeks were significantly higher in patients positive for IF-ANA before IFX and ADA treatment than in the IF-ANA negative group (*P* = 0.034 and *P* = 0.036, respectively). Moreover, high titers of IF-ANA before IFX showed a significant association with HACA appearance within 52 weeks (IF-ANA ≥ 320, odds ratio [OR] = 5.47, 95% confidence intervals [CI] = 1.08–32.8, *P* = 0.040; and IF-ANA ≥ 640, OR = 9.0, 95% CI = 1.50–63.6, *P* = 0.017) (Fig 5A). There was also an association with treatment inefficacy (IF-ANA ≥ 320, OR = 5.47, *P* = 0.040; and IF-ANA ≥ 640, OR = 9.0, *P* = 0.017) (Fig 5D) as well as with treatment discontinuation (IF-ANA ≥ 640, OR = 10.8, *P* = 0.023) (Fig 5G). Higher titers of IF-ANA before IFX were strongly associated with HACA appearance, treatment inefficacy, and discontinuation until 156 weeks of IFX therapy (Fig 5B, 5E and 5H). On the other hand, IF-ANA titer before ADA treatment was not related to AAA appearance, treatment inefficacy, or discontinuation (Fig 5C, 5F and 5I).

## Discussion

This article was an observational study of ANA change in RA patients treated with biologics, and was also incorporated with a study of ADrA in part prospectively to clarify the direct relationship of ANA to ADrA appearance in 121 RA patients treated with TNFi. Total ANA and disease-specific ANA were serially measured by a computer-aided immunofluorescence microscopy system (IF-ANA) and by multiplex flow immunoassay (ANA Screen), respectively. We found that IF-ANA increased during the therapy only in IFX, but not so much in ADA and ETN; both HACA and AAA frequently appeared in patients positive for IF-ANA before therapy, especially positive for that of high titers, and often connected to treatment failure (Fig 1). We compared the characteristics of patients with and without ADrA against IFX or ADA (S1 Table and S2 Table). No significant differences were seen between the patients with and without ADrA. However, as previously reported [1,2], MTX dosage in IFX group or concomitant MTX in ADA group were probably related with immunogenicity (*P* = 0.069 and *P* = 0.060, respectively).

There has been a growing number of reports on the development of systemic autoimmune diseases such as SLE and systemic vasculitis in RA patients treated with TNFi, but the rate was roughly less than 1% [6,7]. Among the 121 cases examined in the present study, there was no onset of lupus during the observation period. Autoantibodies such as ANA and anti-dsDNA Ab have been often newly induced or increased during anti-TNF therapy [9,10], which reportedly lead to poor treatment response [9,11,12]. These reports suggest that anti-TNFi therapy evoked autoimmune reactions and induced the treatment failure of the patients. Ishikawa et al. examined the association between immunogenicity, autoantibody production, and serum cytokine profiles in 57 patients treated with IFX, reporting that patients positive for ADrA (HACA) developed ANA with ≥160 titers, compared with ADrA negative patients, and that anti-DNA Abs significantly increased in the positive patients compared with the negative patients [17]. Thus, they speculated a linkage of development of HACA (immunogenicity) and lupus-like autoantibody production (autoimmunity) along with increased levels of type I interferon. Jani et al. suggested that patients predisposed to developing immunogenicity might also be prone to seroconversion of other autoantibodies [14]. We essentially agree with the opinion, but also think that the opposite may occur as well: patients predisposed to developing autoimmunity may be prone to producing anti-TNFi antibodies.

Here, we clarified that all 30 cases with ADrA were positive for IF-ANA before TNFi while there was no appearance of ADrA over the long term in the 15 patients negative for IF-ANA before anti-TNF therapy (Fig 1). The association between IF-ANA before TNFi treatment and ADrA appearance is noteworthy. Thus, we could predict before TNFi therapy no appearance of ADrA by negative IF-ANA and the appearance of ADrA by positive ANA Screen. Moreover, if the patient had high IF-ANA titers before IFX therapy, LR+ for ADrA appearance increased up to 2.68 and 5.00 in cases of ≥320 or ≥640, respectively. As appearance of ADrA was significantly associated with inefficacy and discontinuation of the treatment, measurement of ANA before TNFi therapy was thought to be useful in routine medical treatment. This is the first report showing the direct association of high ANA titers with HACA appearance in RA patients treated with IFX, although these finding were partly reported in 29 patients with psoriasis by Hoffmann et al. [18]. They reported that HACA-positive patients had significantly higher pretreatment concentrations of dsDNA Ab and higher pretreatment ANA titers than HACA-negative patients (*P* = 0.012 and *P* = 0.075, respectively).

It is reported that long intervals of IFX infusions induce HACA because undetectable serum levels of IFX may be a risk of ADrA and bDMARD dose and treatment interval is critical for ADrA production [1,2]. In our study, the interval of IFX, ADA and ETN were mostly stable during the therapy, and the dosage of IFX, ADA and ETN was not significantly different between the patients with and without ADrA. Although the dosage of TNFi increased in 29 patients treated with IFX and 5 patients treated with ADA, this change was not observed in any patient treated with ETN. However, in patients treated with IFX, 12 among 14 patients (85.5%) positive for ADrA received increased dosage during the therapy, but the dosage was also increased in 17 among 24 patients (70.8%) negative for ADrA. The percentage of patients receiving increased dosage was not significantly different, suggesting that TNFi themselves neutralize and diminish ADrA.

We have shown two additional pieces of evidences suggesting that patients predisposed to developing autoimmunity may be prone to produce anti-TNFi antibodies. First, we could predict the appearance of ADrA by positive ANA Screen (OR: 2.7, *P* = 0.048) and possibly by positive SSA Ab (OR: 3.04, *P* = 0.062) before TNFi therapy. SSA Ab was most frequently observed among 11 components of disease-specific ANA; however, only 15 patients (16.5%) were positive among 91 patients. Therefore, it is difficult to say clearly that SSA Ab test could be a replacement for ANA Screen test, but we consider that presence of SSA Ab was also a risk factor for ADrA appearance as reported [19,20]. Matsudaira et al. reported that SSA Ab is an independent factor associated with insufficient response to TNFi therapy in RA patients but that positive rate and titers of SSA Ab did not change during the therapy, while ANA and dsDNA Ab titers were increased from baseline [19]. Hagiwara et al. mentioned that SSA Ab-positive RA patients treated with IFX showed higher frequency of HACA and seroconversion of ANA [20]. Another piece of evidence is the significant increment in dsDNA Ab titers after therapy in patients treated with either IFX, ADA, or ETN (S3 Fig), suggesting that autoimmunity was enhanced by inhibiting the TNF pathway. However, the DNA Ab titers increased more in ADrA-positive patients than in ADrA negative patients with IFX therapy (S5 Fig), but not with ADA therapy. At least in our study, not only higher pretreatment IF-ANA titers, but also higher IF-ANA titers after IFX therapy, were closely associated with HACA-positivity.

Therefore, it is speculated that predisposition of autoimmunity such as positivity for IF-ANA, SSA Ab, and dsDNA Ab are prone to generate ADrA such as HACA and AAA (immunogenicity), and that the relationship between autoimmunity and immunogenicity was prominent in IFX therapy because HACA production was closely associated with increased IF-ANA and dsDNA Ab titers from baseline. The mechanism was unclear; however, we consider that stronger interaction between immunogenicity and autoimmunity is evoked during IFX therapy than during ADA therapy. This speculation is based on the increase in IF-ANA during IFX therapy, but not so much during ADA, and that IFX was anti-TNF antibodies of chimeric type and stronger immunogenic response to human. However, TNFi themselves not only evoke autoimmunity but also neutralize ADrA. Therefore, increase in the dose and shortening of treatment interval that was often performed in IFX therapy is transiently effective, but possibly accelerates the production of ADrA as well as autoantibodies.

The limitations of this study include the small sample size. In order to make a clear conclusion regarding the relationship between autoimmunity and immunogenicity, it is necessary to increase the number of cases for further examination. Second, the positive rate for IF-ANA before TNFi was roughly 80% and slightly high. Positive titers in our study included staining patterns other than nuclei, such as cytoplasm and centrosome. IF-ANA was measured using a computer-aided immunofluorescent microscopy system (EUROPattern), which had high sensitivity and was assessed on a monitor instead of direct visualization through a microscope. However, the IF-ANA positivity in 40 IF-ANA tested patients among the 42 excluded patients was 75.0% and was not significantly different from the value of 83.5% in the 121 enrolled patients. The IF-ANA positive rate before therapy in 33, 69, and 27 patients treated with tocilizmab, abatacept, and golimumab was 87.9%, 81.7%, and 81.5% respectively, which was almost same as that of our enrolled patients. However, positivity of IF-ANA in 493 RA patients not treated with biologics was 74.6% and lower than that of 83.5% in the 121 enrolled patients (*P =* 0.0145), suggesting high IF-ANA positivity for patients treated with biologics because of their higher disease activity. The second limitation is that our results could be brought about by the highly sensitive IF-ANA assay.

The present study suggests that the presence of IF-ANA before the TNFi therapy is a risk factor for ADrA appearance as well as for treatment inefficacy. Measuring IF-ANA could be useful to make an appropriate choice of IFX, ADA, and ETN. If IF-ANA is negative, any of the three TNFi can be used without ADrA. It may be better to avoid IFX and probably ADA for patients with ANA of high titers. We believe that this study is novel in suggesting that the measurement of IF-ANA before TNFi in RA may help predict secondary failure and adverse events through estimation of ADrA appearance and in directly showing that the interaction of immunogenicity with autoimmunity could be brought during anti-TNF therapy.

## Conclusion

Our findings suggest that the presence of IF-ANA before TNFi therapy is a risk factor for ADrA appearance as well as for treatment inefficacy. Fourteen patients (36.8%) treated with IFX and 16 patients (30.2%) with ADA turned positive for ADrA, all of whom were positive for IF-ANA before the therapy. In contrast, none turned positive for ADrA in 15 patients negative for IF-ANA before therapy (6 with IFX and 9 with ADA). The presence of IF-ANA before anti-TNF therapy may be a risk factor for the appearance of ADrA. In patients treated with IFX, high titers of IF-ANA before and after therapy correlated with ADrA appearance and could predict ADrA appearance and possibly secondary failure. Therefore, measuring IF-ANA before TNFi therapy may be useful for appropriately selecting the TNFi. Among the three TNFi, IF-ANA increased especially during IFX therapy, and only IFX showed a close relationship between IF-ANA and ADrA appearance, suggesting interaction of immunogenicity with autoimmunity. Since the number of cases examined in this study was small, it is necessary to increase the number of cases for further examination.

## Supporting information

S1 Fig Relevance of ANA Screen presence before anti-TNF therapy to appearance of ADrA. The rate of ADrA appearance between 0–52 weeks was compared with ANA Screen presence or absence before anti-TNF therapy. The percentage and absolute number of each group are indicated above the bar in each graph. Fisher’s exact test was used for comparison. ADrA, anti-drug antibodies; IFX, infliximab; and ADA, adalimumab.
(TIF)

S2 Fig ANA Screen, 11 components of disease-specific ANA before TNFi therapy, and ADrA appearance. The absolute number of patients positive for ANA Screen, and each of the 11 items of disease-specific ANA before TNFi (IFX + ADA) therapy (at 0–52 weeks) are shown in the bar graphs (n = 91). Numbers in the bar graphs colored in gray indicate the patients who became positive for ADrA, and numbers outside the bar graphs indicate the total number of patients. IFX, infliximab; ADA, adalimumab; ADrA, anti-drug antibodies.
(TIF)

S3 Fig Changes in the titers of dsDNA Ab after anti-TNF therapy. Titers of dsDNA Ab were shown before and after anti-TNF therapy in patients treated with TNFi (IFX, 0–52 weeks and 0–156 weeks; ADA, 0–52 weeks; and ETN, 0–52 weeks). Each fine line shows a single patient, and the bold lines show the average titers as mean ± SEM. In one patient in the ADA-treated group, the titer was 17 IU/ml before the therapy and increased to 44 IU/ml after the therapy. Wilcoxon signed rank test was used for comparison. IFX, infliximab; ADA, adalimumab; and ETN, etanercept.
(TIF)

S4 Fig Relevance of IF-ANA increase after anti-TNF therapy to the appearance of ADrA. The rate of ADrA positive was compared by IF-ANA increased (↑) or not increased (→ or ↓) after anti-TNF therapy. The percentages and absolute numbers of each group of patients are indicated above the bar graphs. The Fisher’s exact test was used for comparison. ADrA, anti-drug antibodies; IFX, infliximab; ADA, adalimumab.
(TIF)

S5 Fig Comparison of DNA Ab titers before and after IFX therapy between HACA-positive and negative patients. Each line shows a single patient treated with IFX (0–156 weeks). Solid and dashed lines show patients positive and negative for HACA, respectively. The bold lines show the average titers as the mean ± SEM. The titers of dsDNA Ab increased more significantly in the patients positive for HACA than in those negative. Two patients whose titers of dsDNA Ab became ≥10 IU/mL after therapy were judged as having seroconversion of dsDNA Ab and were shown positive for HACA at the same time. The titers before and after IFX therapy in the group positive or negative for HACA were noted as the mean ± SEM under the line graph. The Mann-Whitney U test was used for inter-group comparison. ns: not significant; *: *P* = 0.014.
(TIF)

S1 Table Characteristics of 38 RA patients treated with IFX, according to the presence or absence of anti-drug antibodies. (TIF)

S2 Table Characteristics of 53 RA patients treated with ADA, according to the presence or absence of anti-drug antibodies. (TIF)

## References

[1] Kalden JR, Schulze-Koops H. Immunogenicity and loss of response to TNF inhibitors: Implications for rheumatoid arthritis treatment Vol. 13, Nature Reviews Rheumatology. Nature Publishing Group; 2017 p. 707–18. 10.1038/nrrheum.2017.187 . 29158574

[2] Garcês S, Demengeot J, Benito-Garcia E. The immunogenicity of anti-TNF therapy in immune-mediated inflammatory diseases: A systematic review of the literature with a meta-analysis. Ann Rheum Dis. 2013;72(12):1947–1955. 10.1136/annrheumdis-2012-202220 . 23223420

[3] Moots RJ, Xavier RM, Mok CC, Rahman MU, Tsai W-C, Al-Maini MH, et al The impact of anti-drug antibodies on drug concentrations and clinical outcomes in rheumatoid arthritis patients treated with adalimumab, etanercept, or infliximab: Results from a multinational, real-world clinical practice, non-interventional study. PLoS One. 2017 4 1; 12(4):e0175207 10.1371/journal.pone.0175207 . 28448562 PMC5407581

[4] Jani M, Chinoy H, Warren RB, Griffiths CEM, Plant D, Fu B, et al Clinical utility of random anti-tumor necrosis factor drug-level testing and measurement of antidrug antibodies on the long-term treatment response in rheumatoid arthritis. Arthritis Rheumatol. 2015 8 1; 67(8):2011–9. 10.1002/art.39169 ; PMCID: PMC4843946. 26109489 PMC4843946

[5] Maneiro JR, Salgado E, Gomez-Reino JJ. Immunogenicity of monoclonal antibodies against tumor necrosis factor used in chronic immune-mediated inflammatory conditions: Systematic review and meta-analysis. JAMA Intern Med. 2013;173(15):1416–1428. 10.1001/jamainternmed.2013.7430 . 23797343

[6] De Bandt M, Sibilia J, Le Loët X, Prouzeau S, Fautrel B, Marcelli C, et al Systemic lupus erythematosus induced by anti-tumour necrosis factor alpha therapy: a French national survey. Arthritis Res Ther. 2005; 7(3):R545–51. 10.1186/ar1715 ; PMCID: PMC1174953. 15899041 PMC1174953

[7] Ramos-Casals M, Brito-Zerón P, Muñoz S, Soria N, Galiana D, Bertolaccini L, et al Autoimmune diseases induced by TNF-targeted therapies: analysis of 233 cases. Medicine (Baltimore). 2007 7; 86(4):242–51. 10.1097/MD.0b013e3181441a68 . 17632266

[8] Wetter DA, Davis MDP. Lupus-like syndrome attributable to anti-tumor necrosis factor α therapy in 14 patients during an 8-year period at mayo clinic. Mayo Clin Proc. 2009;84(11):979–984. 10.4065/84.11.979 . 19880688 PMC2770909

[9] Takase K, Horton SC, Ganesha A, Das S, McHugh A, Emery P, et al What is the utility of routine ANA testing in predicting development of biological DMARD-induced lupus and vasculitis in patients with rheumatoid arthritis? Data from a single-centre cohort. Ann Rheum Dis. 2014 9; 73(9):1695–9. 10.1136/annrheumdis-2014-205318 . 24854356

[10] Vaz J, Fernandes V, Nogueira F, Arnobio A, Levy R. Infliximab-induced autoantibodies: a multicenter study. Clin Rheumatol. 2016;35(2):352–4. 10.1007/s10067-015-3140-6 . 26676808

[11] Yukawa N, Fujii T, Kondo-Ishikawa S, Yoshifuji H, Kawabata D, Nojima T, et al Correlation of antinuclear antibody and anti-double-stranded DNA antibody with clinical response to infliximab in patients with rheumatoid arthritis: a retrospective clinical study. Arthritis Res Ther. 2011 12 22; 13(6):R213 10.1186/ar3546 . 22192852 PMC3334666

[12] Ishikawa Y, Hashimoto M, Ito H, Tanaka M, Yukawa N, Fujii T, et al Anti-nuclear antibody development is associated with poor treatment response to biological disease-modifying anti-rheumatic drugs in patients with rheumatoid arthritis. Semin Arthritis Rheum. 2019 10 1; 49(2):204–10. 10.1016/j.semarthrit.2019.02.003 . 30803720

[13] Hoxha A, Calligaro A, Tonello M, Ramonda R, Carletto A, Paolazzi G, et al The clinical relevance of early anti-adalimumab antibodies detection in rheumatoid arthritis, ankylosing spondylitis and psoriatic arthritis: A prospective multicentre study. Jt bone spine. 2016 3 1; 83(2):167–71. 10.1016/j.jbspin.2015.04.020 . 26750762

[14] Jani M, Dixon WG, Chinoy H. Drug safety and immunogenicity of tumour necrosis factor inhibitors: the story so far. Rheumatology (Oxford). 2018; 57(11):1896–907. 10.1093/rheumatology/kex434 ; PMCID: PMC6199532. 29325166 PMC6199532

[15] Wells G, Becker J-C, Teng J, Dougados M, Schiff M, Smolen J, et al Validation of the 28-joint Disease Activity Score (DAS28) and European League Against Rheumatism response criteria based on C-reactive protein against disease progression in patients with rheumatoid arthritis, and comparison with the DAS28 based on erythrocyte sedimentation rate. Ann Rheum Dis. 2009 6; 68(6):954–60. 10.1136/ard.2007.084459 ; PMCID: PMC2674547. 18490431 PMC2674547

[16] Voigt J, Krause C, Rohwäder E, Saschenbrecker S, Hahn M, Danckwardt M, et al Automated indirect immunofluorescence evaluation of antinuclear autoantibodies on HEp-2 cells. Clin Dev Immunol. 2012; 2012:651058 10.1155/2012/651058 . 23251220 PMC3502836

[17] Ishikawa Y, Fujii T, Ishikawa SK, Yukawa N, Hashimoto M, Furu M, et al Immunogenicity and Lupus-Like Autoantibody Production Can Be Linked to Each Other along With Type I Interferon Production in Patients with Rheumatoid Arthritis Treated With Infliximab: A Retrospective Study of a Single Center Cohort. PLoS One. 2016 9 1; 11(9):e0162896 10.1371/journal.pone.0162896 ; PMCID: PMC5028026. 27643491 PMC5028026

[18] Hoffmann JHO, Hartmann M, Enk AH, Hadaschik EN. Autoantibodies in psoriasis as predictors for loss of response and anti-infliximab antibody induction. Br J Dermatol. 2011 12; 165(6):1355–8. 10.1111/j.1365-2133.2011.10555.x . 21801160

[19] Matsudaira R, Tamura N, Sekiya F, Ogasawara M, Yamanaka K, Takasaki Y. Anti-Ro/SSA antibodies are an independent factor associated with an insufficient response to tumor necrosis factor inhibitors in patients with rheumatoid arthritis. J Rheumatol. 2011;38(11):2346–54. 10.3899/jrheum.101295 . 21965648

[20] Hagiwara S, Tsuboi H, Honda F, Takahashi H, Kurata I, Ohyama A, et al Association of anti-Ro/SSA antibody with response to biologics in patients with rheumatoid arthritis. Mod Rheumatol. 2016;26(6):857–62. 10.3109/14397595.2016.1153567 . 26873159

