# Supplementary figures and images for: Presence of anti-nuclear antibodies is a risk factor for the appearance of anti-drug antibodies during infliximab or adalimumab therapy in patients with rheumatoid arthritis
Source: PLoS One. 2020 Dec 14;15(12):e0243729. doi: 10.1371/journal.pone.0243729 (PMC7735569; doi:10.1371/journal.pone.0243729)

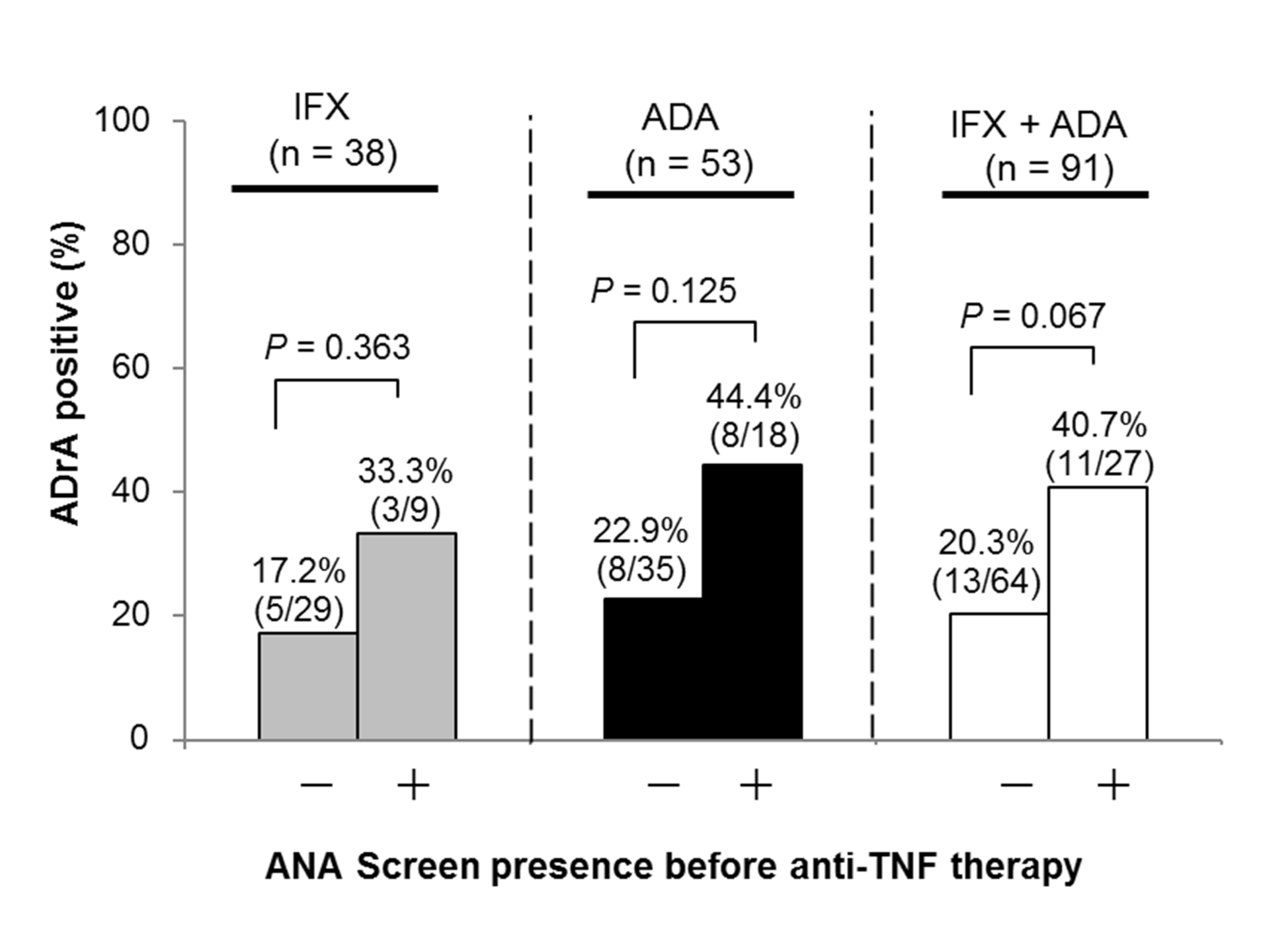

Supplement: S1 Fig — The rate of ADrA appearance between 0–52 weeks was compared with ANA Screen presence or absence before anti-TNF therapy. The percentage and absolute number of each group are indicated above the bar in each graph. Fisher’s exact test was used for comparison. ADrA, anti-drug antibodies; IFX, infliximab; and ADA, adalimumab. (TIF) [file pone.0243729.s001.tif]

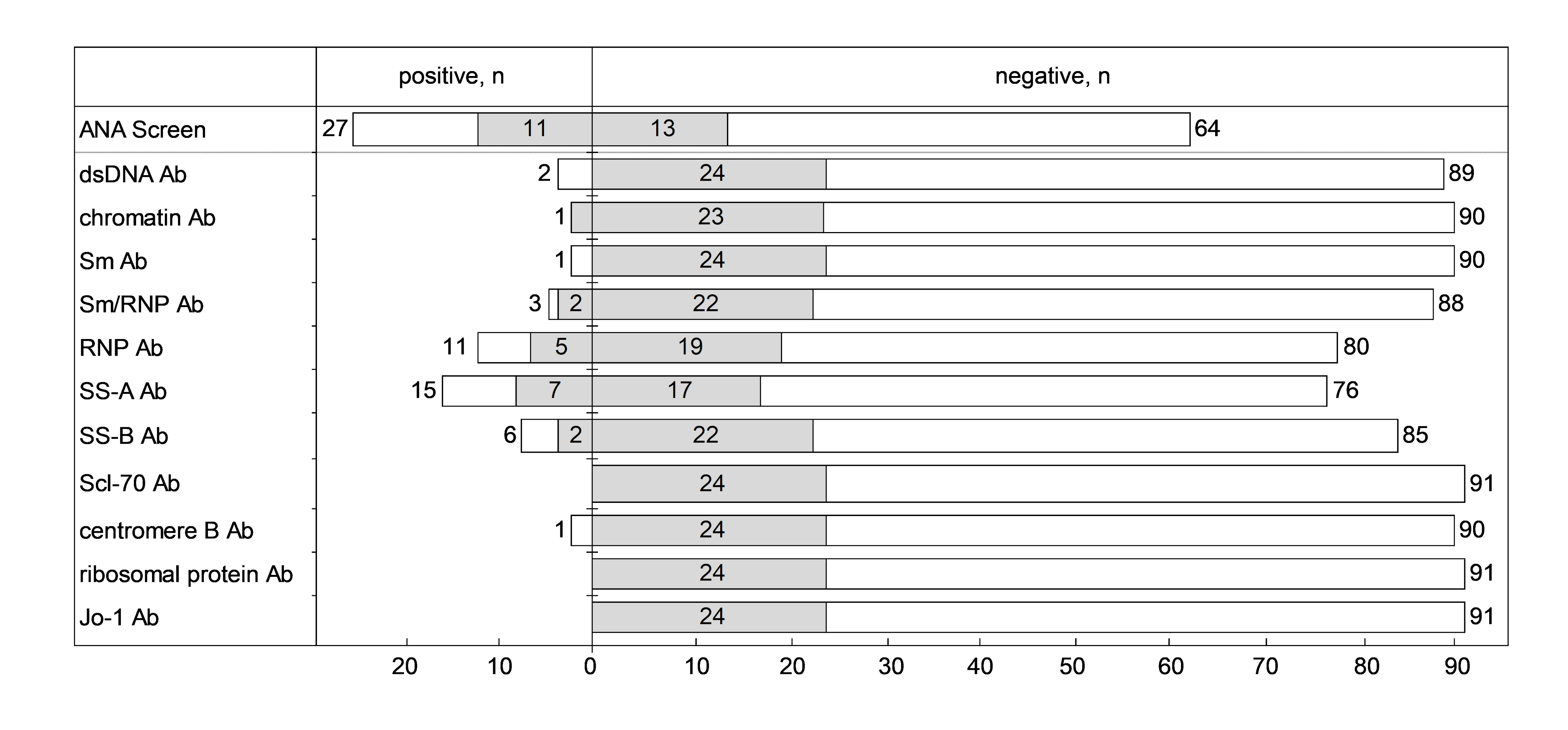

Supplement: S2 Fig — The absolute number of patients positive for ANA Screen, and each of the 11 items of disease-specific ANA before TNFi (IFX + ADA) therapy (at 0–52 weeks) are shown in the bar graphs (n = 91). Numbers in the bar graphs colored in gray indicate the patients who became positive for ADrA, and numbers outside the bar graphs indicate the total number of patients. IFX, infliximab; ADA, adalimumab; ADrA, anti-drug antibodies. (TIF) [file pone.0243729.s002.tif]

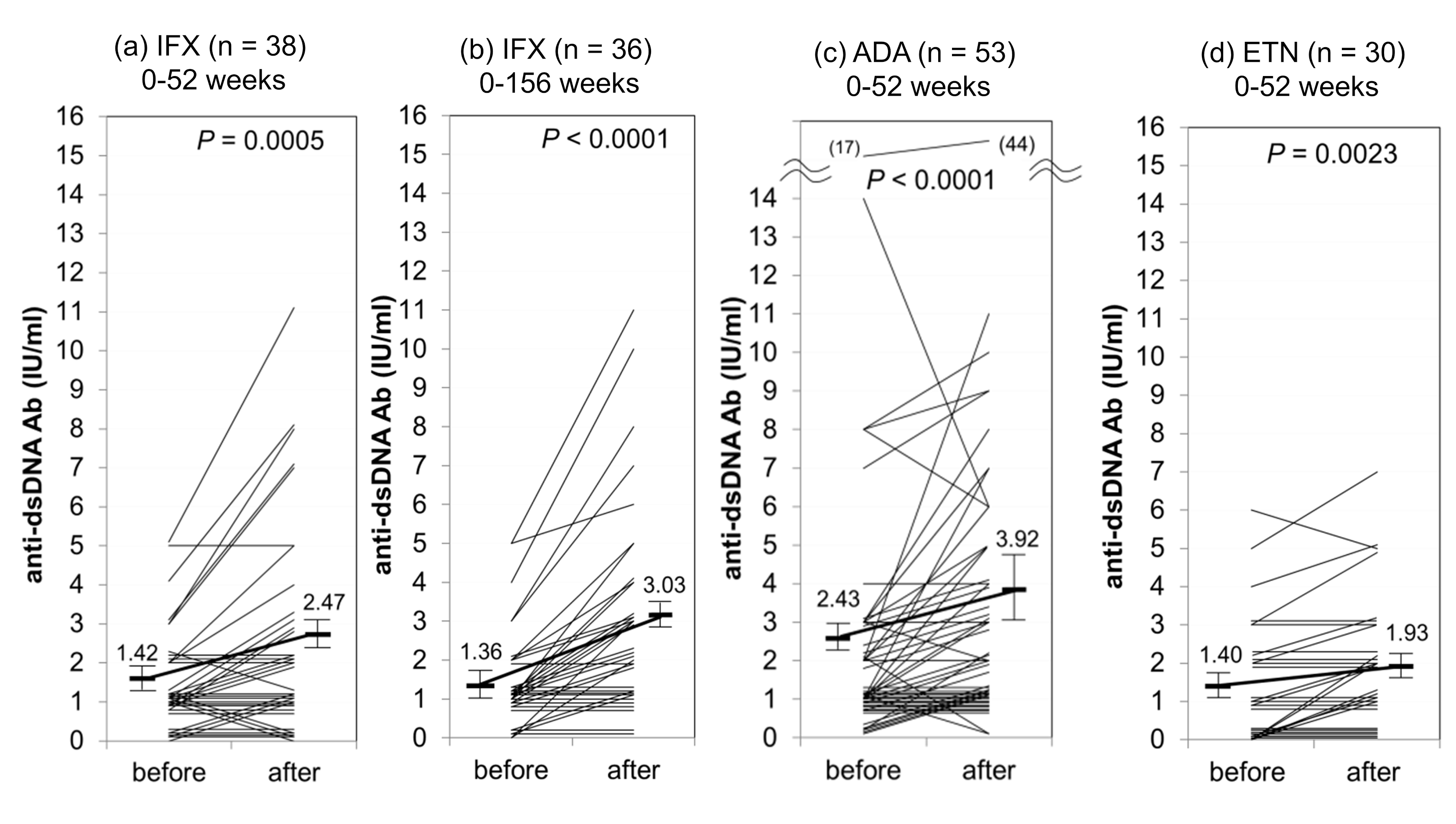

Supplement: S3 Fig — Titers of dsDNA Ab were shown before and after anti-TNF therapy in patients treated with TNFi (IFX, 0–52 weeks and 0–156 weeks; ADA, 0–52 weeks; and ETN, 0–52 weeks). Each fine line shows a single patient, and the bold lines show the average titers as mean ± SEM. In one patient in the ADA-treated group, the titer was 17 IU/ml before the therapy and increased to 44 IU/ml after the therapy. Wilcoxon signed rank test was used for comparison. IFX, infliximab; ADA, adalimumab; and ETN, etanercept. (TIF) [file pone.0243729.s003.tif]

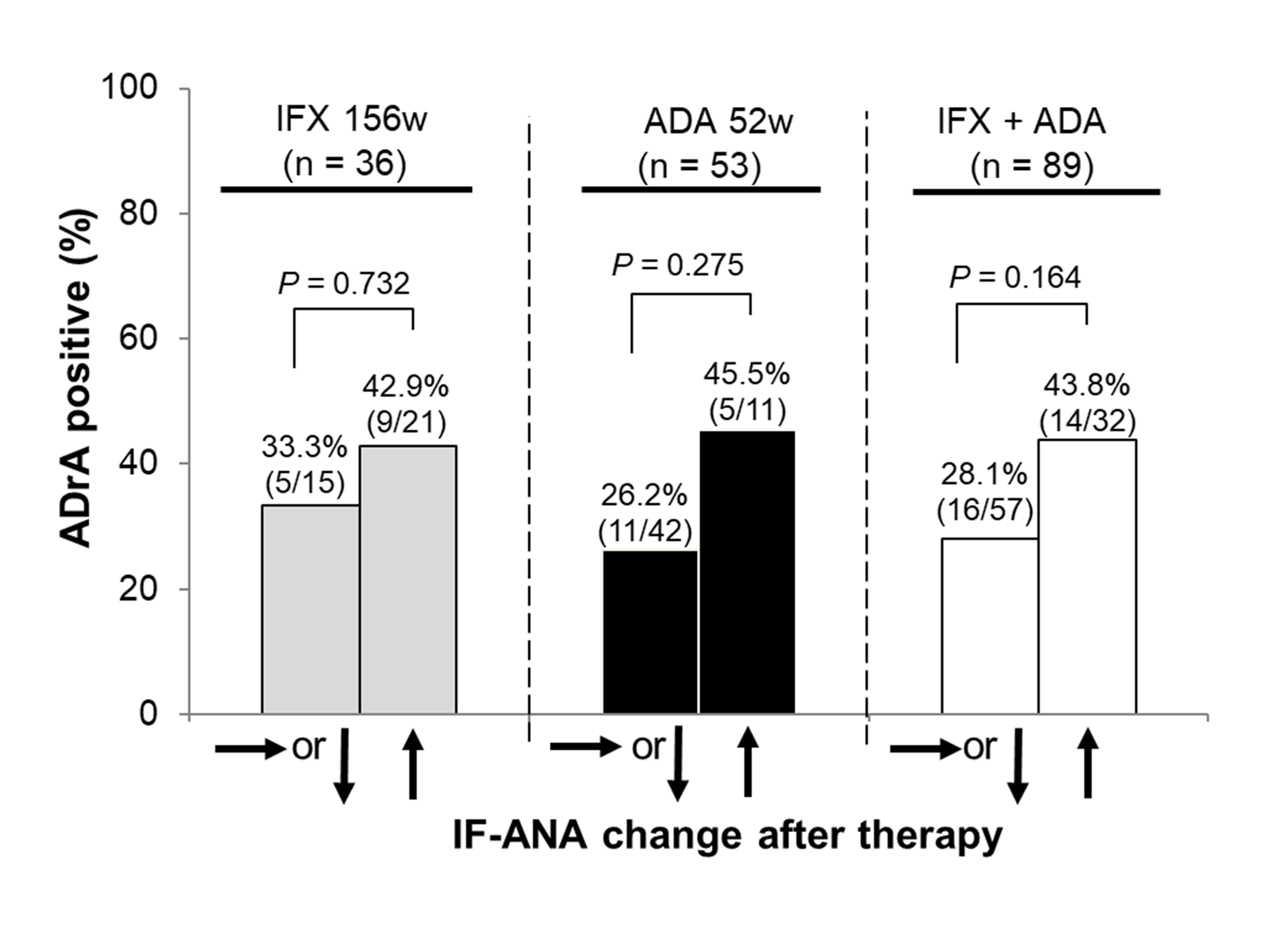

Supplement: S4 Fig — The rate of ADrA positive was compared by IF-ANA increased (↑) or not increased (→ or ↓) after anti-TNF therapy. The percentages and absolute numbers of each group of patients are indicated above the bar graphs. The Fisher’s exact test was used for comparison. ADrA, anti-drug antibodies; IFX, infliximab; ADA, adalimumab. (TIF) [file pone.0243729.s004.tif]

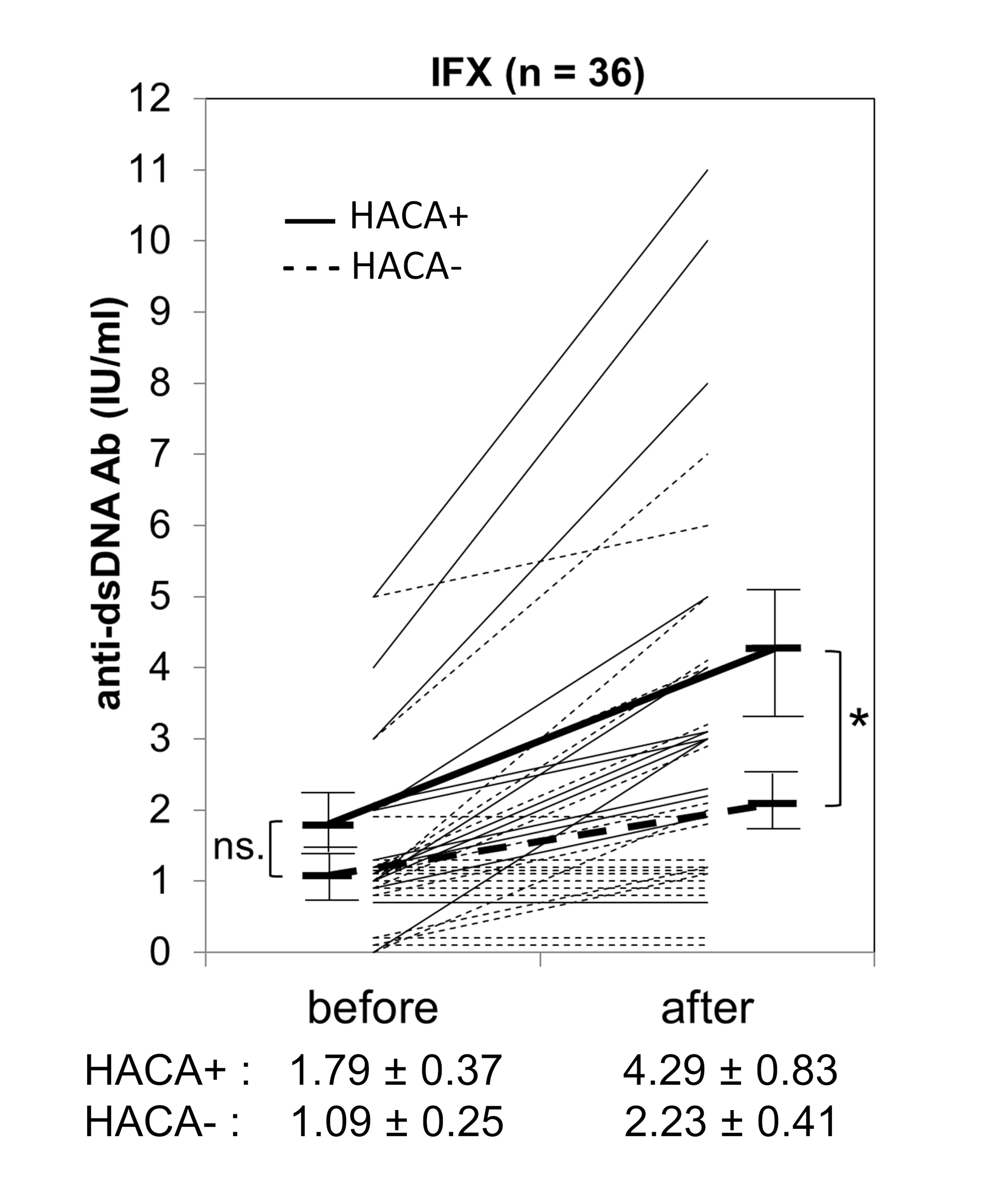

Supplement: S5 Fig — Each line shows a single patient treated with IFX (0–156 weeks). Solid and dashed lines show patients positive and negative for HACA, respectively. The bold lines show the average titers as the mean ± SEM. The titers of dsDNA Ab increased more significantly in the patients positive for HACA than in those negative. Two patients whose titers of dsDNA Ab became ≥10 IU/mL after therapy were judged as having seroconversion of dsDNA Ab and were shown positive for HACA at the same time. The titers before and after IFX therapy in the group positive or negative for HACA were noted as the mean ± SEM under the line graph. The Mann-Whitney U test was used for inter-group comparison. ns: not significant; *: P = 0.014. (TIF) [file pone.0243729.s005.tif]

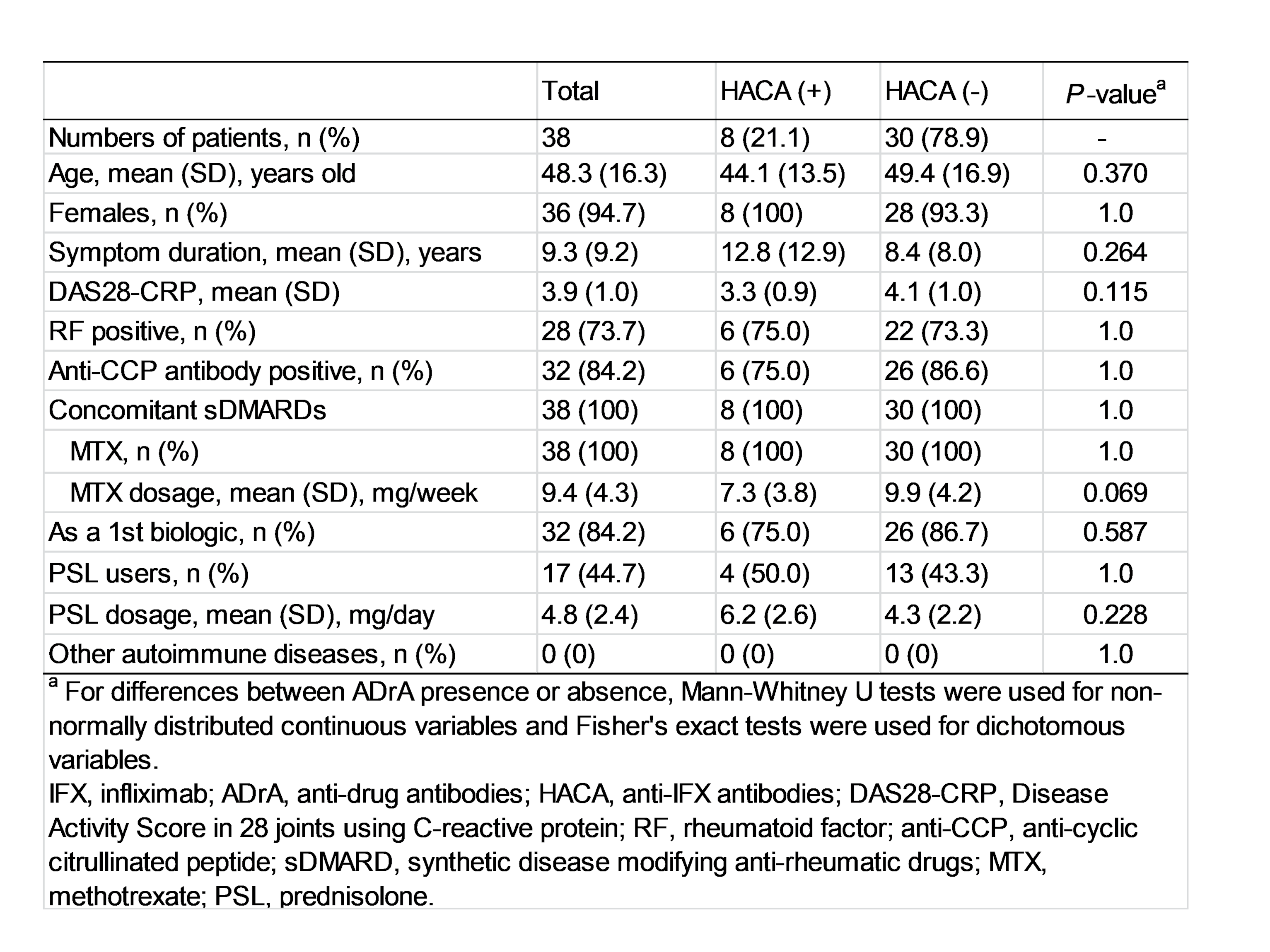

Supplement: S1 Table — (TIF) [file pone.0243729.s006.tif]

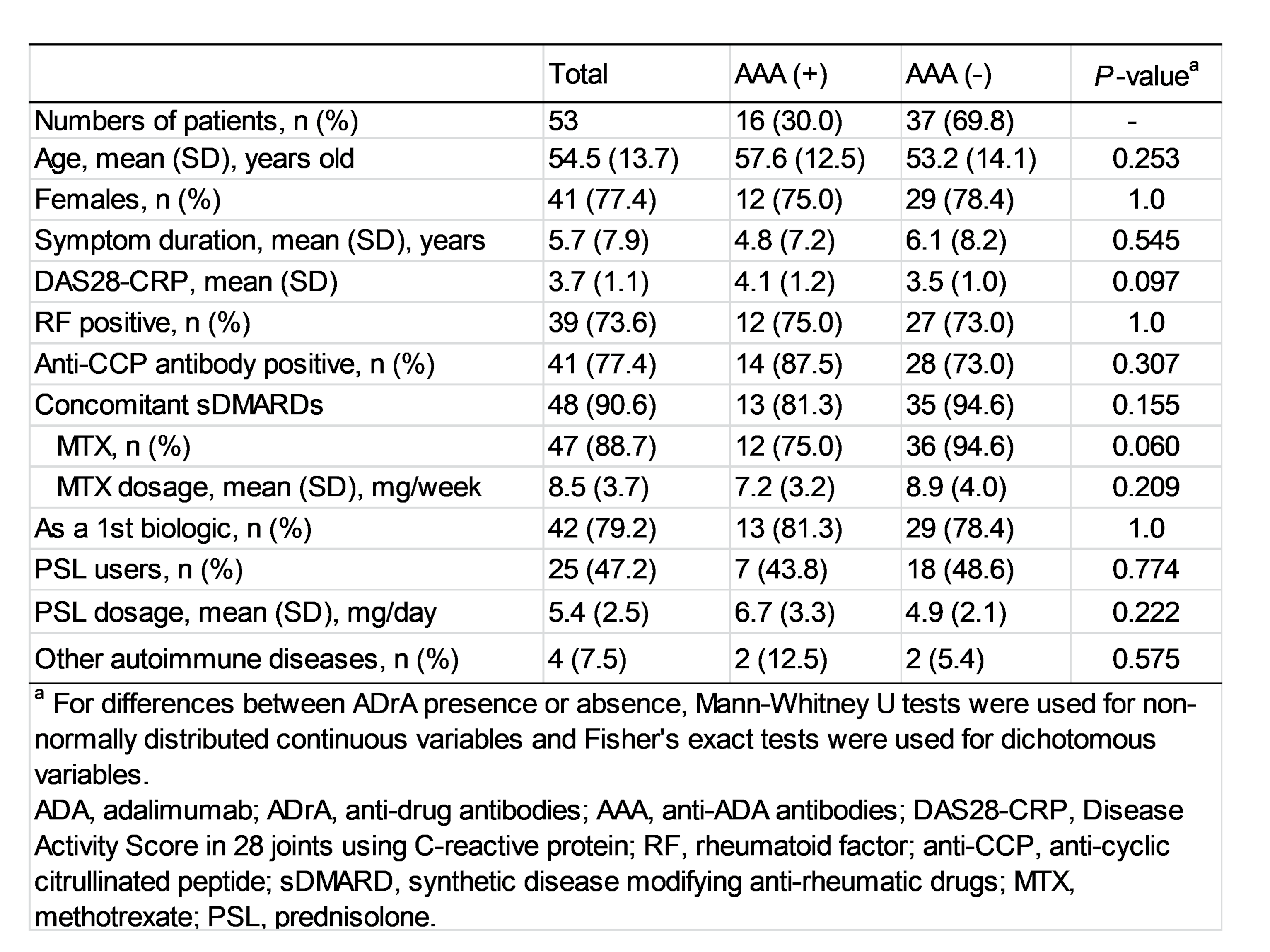

Supplement: S2 Table — (TIF) [file pone.0243729.s007.tif]
